# Non-pharmacological treatment in difficult-to-treat rheumatoid arthritis

**DOI:** 10.3389/fmed.2022.991677

**Published:** 2022-08-29

**Authors:** Judit Majnik, Noémi Császár-Nagy, Georgina Böcskei, Tamás Bender, György Nagy

**Affiliations:** ^1^Department of Rheumatology and Clinical Immunology, Semmelweis University, Budapest, Hungary; ^2^Hospital of the Hospitaller Order of Saint John of God, Budapest, Hungary; ^3^Department of Public Management and Information Technology, Faculty of Science of Public Governance and Administration, National University of Public Service, Budapest, Hungary; ^4^Department of Internal Medicine and Oncology, Semmelweis University, Budapest, Hungary; ^5^Heart and Vascular Center, Semmelweis University, Budapest, Hungary; ^6^Department of Genetics, Cell- and Immunobiology, Semmelweis University, Budapest, Hungary

**Keywords:** difficult-to-treat, rheumatoid arthritis, non-pharmacological, treatment, exercise, psychotherapy, physiotherapy

## Abstract

Although the management of rheumatoid arthritis (RA) has improved remarkably with new pharmacological therapies, there is still a significant part of patients not reaching treatment goals. Difficult-to-treat RA (D2TRA) is a complex entity involving several factors apart from persistent inflammation, thereafter requiring a holistic management approach. As pharmacological treatment options are often limited in D2TRA, the need for non-pharmacological treatments (NPT) is even more pronounced. The mechanism of action of non-pharmacological treatments is not well investigated, NPTs seem to have a complex, holistic effect including the immune, neural and endocrine system, which can have a significant additive benefit together with targeted pharmacotherapies in the treatment of D2TRA. In this review we summarize the current knowledge on different NPT in rheumatoid arthritis, and we propose a NPT plan to follow when managing D2TRA patients.

## Introduction

Targeted therapies have revolutionized the treatment of rheumatoid arthritis (RA), and disease remission and prevention of joint destruction has become a realistic goal. Before the widespread use of the new pharmacological therapies, non-pharmacological interventions had a more pronounced role in RA treatment, but in the last decades investigations in this field are less frequent.

The EULAR recommendations and the ACR guideline for the management of RA are focusing on pharmacological therapies based on synthetic and biological DMARDs (1, 2), but several EULAR recommendations include elements regarding different non-pharmacological therapeutic alternatives partly applicable in RA patients (3–6). In 2021 a EULAR Task Force published the definition of difficult-to-treat rheumatoid arthritis (D2TRA) consisting of three main points: (1) failure of at least two biological or targeted synthetic DMARDS; (2) presence of active/progressive disease; and (3) problematic management perceived by the rheumatologist or the patient (7).

**FIGURE 1:**
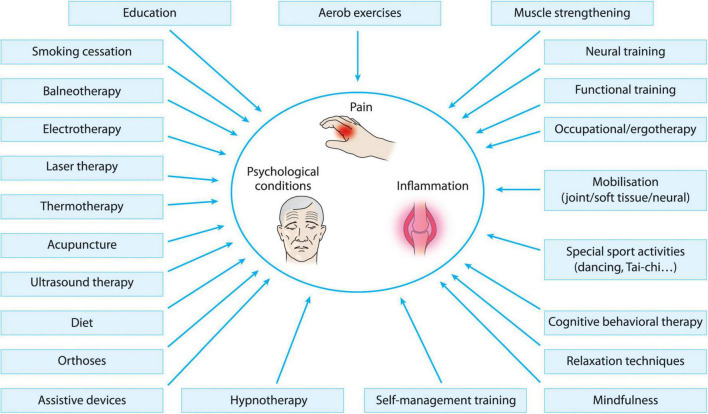
Different non-pharmacological treatment options in D2TRA.

In addition to new medications, the problematic management of D2TRA is highlighting the need for additional therapeutic strategies for reaching treatment goals and attaining a better quality of life in RA. In all RA patients but even more pronounced in D2TRA, drug-intolerance or inefficacy, pain, work disability, psychological and social complications raise our increasing attention to non-pharmacological therapies targeting several of these multiple disease aspects (Figure 1). Pharmacological and non-pharmacological therapies have an additional, synergistic effect in the treatment of RA and D2TRA, and non-pharmacological interventions can support the success of pharmacological therapies. The EULAR points to consider for the management of D2TRA (8, 9) include elements concerning non-pharmacological interventions, which have an increasingly recognized role in D2TRA.

The aim of this review is to summarize the recent knowledge about different non-pharmacological interventions in RA, to appoint those that could be of specific interest in D2TRA, and to suggest a practical structure of non-pharmacological treatment approach in D2TRA. The literature regarding non-pharmacological interventions is wide, however studies focusing on D2TRA, due to the recent establishment of D2TRA criteria are yet lacking. Data obtained in other groups of RA patients with established, active disease, long-lasting symptoms can be applied partly on D2TRA patients, and this review is focusing on these study results. In the literature search, under established RA we meant patients with clinical symptoms duration greater than 2 years (when defined in the study), and excluded studies about early RA or non-differentiated arthritis, under active disease we meant elevated DAS28 score (>3.2) or pain level (>40% on VAS) or elevated number of swollen or tender joints, and under long-lasting symptoms mainly pain, disability or functional limitations and comorbidities e.g., depression. The detailed summary of the reviewed literature is available in Supplementary Tables 1–3.

## Exercise therapy

The positive effect of physical activity (PA) in inflammatory rheumatic diseases is well documented (4), and a 2019 review on different non-pharmacological treatment modalities in RA concluded that only exercise/physical activity interventions appeared to be effective in reducing the global impact of disease and quality of life (10). Exercise training programs seem to have the greatest benefit in those RA patients who are older, more inflamed and less fit (11), characteristics applicable to many D2TRA patients, thus emphasizing the role of exercise therapy in this patient group.

Rheumatoid arthritis patients with more active disease are prone to be physically less active and have more sedentary lifestyle (12), which also highlight the importance of maintaining physical activity in D2TRA. For RA patients with high disease activity, high rate of disability and long-standing disease, a person-centered, individualized exercise program can be helpful in improving self-efficacy and physical activity on the long term (13).

Summary of RCTs, Cochrane reviews and meta-analyses is presented in Supplementary Table 1. The aim of exercise interventions in RA are multiple including increase of endurance, agility, increase of muscle strength and mass, decrease of joint/soft tissue pain and swelling, improvement of musculoskeletal function, decrease of cardiovascular risk, improvement of self-efficacy and quality of life. There is much evidence, although mostly of low or moderate quality about the positive effect of dynamic, aerobic exercise programs, muscle strengthening exercises in RA patients on pain, function, fatigue, quality of life (14–18), and some data about positive effect on DAS28 score and inflammatory markers (ESR) (19). The benefits of Yoga or Tai-Chi in RA are uncertain or of low evidence (20–22). Regarding mobilization and neural techniques in RA there are also some trials, mainly showing an effect on pain (23, 24). As high load resistance training is not always feasible in active RA patients, low load or non-resistance training (25) or water-based exercises (26, 27) appear to be good alternatives.

Physical activity is important not only for musculoskeletal symptoms of RA, but also for several comorbidities, which may be more predominant in the D2TRA group. Physically inactive RA patients have significantly worse CVD risk profile (28), and high CVD risk RA patients may get substantial benefits from a physical activity program (29). Upper- and lower extremity strengthening exercise programs improved not only muscle strength but also mental health domain scores (30), and walking-based physical activity showed improvements in sleep duration and sleep quality in RA (31), although the latter studies were conducted in less active RA patients, thus further investigations are needed to clarify this impact in active, D2TRA patients.

It is a central question of all exercise programs how to achieve long-term persistency. This question points toward both educational and psychological techniques, showing the integrity of non-pharmacological treatment modalities. The transition from supervised to independent exercise and the long-term sustainment of physical activity can be supported by personal motivation interventions (32) and internet based techniques (33, 34). Several studies examined different home exercise programs. A SLR on home-based PA interventions in autoimmune rheumatic diseases including RA patients showed improved quality of life and functional capacity, reduced pain and disease activity similar to center-based interventions (35). Adherence and efficacy in home exercise programs can be improved with special strategies including education, self-management and training sessions (17). The importance of home-based programs has become even more emphasized due to the restrictions of COVID pandemic, and physical activity programs are showed to have positive effect on mental state and better vitality in RA (36).

## Psychological interventions

In RA, the prevalence of different psychological problems like anxiety and depression is high (37), and even more pronounced in D2TRA (38). Depression was found to be 2–3 times more common in RA patients (39), it is considered as one of the most common comorbidity of RA (40) with common points in cytokine pattern (41), and there seems to be a bidirectional relationship between pain and depression (42).

Several psychological interventions have been studied in the treatment of RA (Supplementary Table 2). The main techniques are (1) education techniques (self-management training, coping skills training, modular behavioral education, patient education), (2) stress management and basic psychotherapies (relaxation techniques, counseling, supportive therapy, mindfulness, self-regulation therapy), (3) specific psychotherapies like cognitive-behavioral therapy (CBT), emotional disclosure (ED), hypnotherapy.

Education and self-management programs are widely used and accepted methods in RA (3, 5, 32, 43, 44), and even internet-based self-management programs have proved to be beneficial (45, 46). Mindfulness-based techniques are easy to perform, do not necessarily need the intervention of a psychotherapist, and are shown to have several beneficial effect on pain-related catastrophizing, fatigue, morning disability (47), pain and even DAS28-CRP score (48). Mindfulness-based group interventions were also shown to be effective on RA self-efficacy, pain, fatigue and overall well-being (49). Cognitive behavioral therapy was examined in RA patients in several study settings, it ameliorated pain, fatigue, function, tender joint count, self-efficacy (44, 50–53), and even internet-based therapy resulted in long persistent improvement of quality of life or tender joint count (46, 54, 55). Emotional disclosure has shown some positive effect in RA, but mostly mixed results have been found, this method seems to be less effective and needs special caution (51, 56).

## Physiotherapy and balneotherapy

Among non-pharmacological treatment options in RA several physiotherapy and balneotherapy modalities have been studied. There are some positive results with laser acupuncture (57), underwater ultrasound (US) therapy (58, 59), transelectrical nerve stimulation (TENS) (60), transcutan stimulation of the cervical vagal nerve (61) and neuromuscular electrical stimulation (62–64). Cryotherapy has been studied in several groups of RA patients, both whole body cryostimulation and local cryotherapy had positive effects on clinical and laboratory parameters (65–67), and can be combined with physical activity with good effect on pain and disease activity (68).

Balneotherapy is widely used in musculoskeletal diseases and the effect in RA has been studied with some positive results, although mainly before the widespread use of biological therapies, thereafter the results should be interpreted with caution in D2TRA. Most positive effects were found with radon containing spa (69, 70), and sulphur spa therapy (71). Several studies about dead sea mud therapies have been published with positive results (72–74).

## Dietary interventions

Different diets and nutritional supplements have been studied in inflammatory rheumatic diseases including RA. Mediterranean diet, especially when combined with physical exercise showed some effect on quality of life (75–77), although some results were obtained in RA patients with lower disease activity. Vitamin D supplementation is beneficial in patients with RA, with positive effects on disease activity (78) and comorbidities e.g., osteoporosis. Fish oil supplements and herbal therapies like gamma-linolenic acid containing seeds or Tripterygium wilfordii (thunder god vine—caution with potential toxic side effects) showed some beneficial clinical effects (79, 80), and there are some data about the potential benefits of probiotic supplementation in RA (81). Obesity can be a contributing factor in D2TRA, and case series, retrospective and pilot studies point toward the beneficial effect of weight loss on disease activity, physical functioning, eating behaviors and pain in RA (82–84). Nevertheless caution is needed with weight loss interventions, as lower BMI has been showed to be associated with increased mortality in RA (85, 86), which emphasizes the importance of personalized dietary programs.

## Other non-pharmacological therapies

Several other non-pharmacological treatment options have been studied in RA patients, but evidence is scarce. Acupuncture showed some benefit on RA activity (87–91), and occupational therapy improved functional and work-related outcomes (92). Although smoking is a known risk factor for RA and for more aggressive disease and smokers are likely to respond less to some but not all TNF antagonists (93, 94), data are scarce about the effect of smoking cessation on disease activity (95). Orthoses and assistive devices are occasionally used by RA patients with some evidence of beneficial effect (96–98), but hand splints can even diminish grip strength (97) thus caution and personalized use is required.

Although out of the scope of this review, several surgical interventions should be mentioned among non-pharmacological therapies in RA (synovectomy with open or arthroscopic surgery or radiosynovectomy with intraarticular isotope injections, reconstructive surgery of ligaments, tendons, joint replacement/endoprosthesis intervention), all of them might be beneficial in D2TRA patients with joint destructions and disabilities. Another important issue is the access of social aid for D2TRA patients, and health care professionals have a role in helping with access to patient organizations, disability benefits, community support.

## Proposed non-pharmacological treatment strategy in D2TRA

**FIGURE 2:**
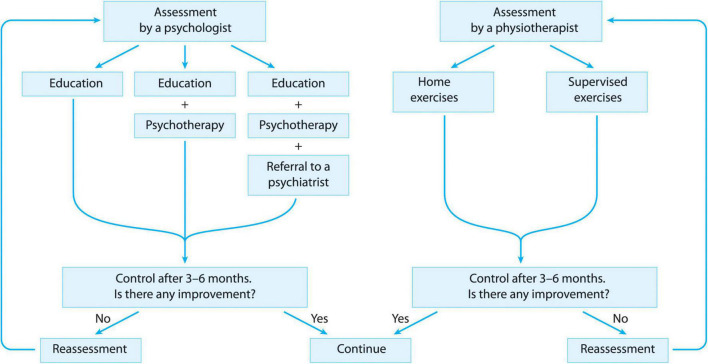
Proposed structure of non-pharmacological treatment in D2TRA.

Based on the evidence found in the literature, we elaborated a NPT approach for D2TRA patients. We suggest that every D2TRA patient should receive an individualized non-pharmacological therapy (NPT) program (Figure 2). The two basic elements are a regular psychologic/educational help and a regular help in sustained physical training including aerobic and strengthening exercises. For this purpose, in parallel with the rheumatologist’s examination, every D2TRA patient should undergo a detailed examination by a psychologist and a physiotherapist, and reassessment should take place every 3/6 months until the patient meets the criteria of remission or low disease activity.

According to the psychologist’s decision, every D2TRA patient can be enrolled in one of the following three intervention categories. No 1: Patients not in need of psychiatric examination or psychotherapy should receive regular educational sessions helping compliance and lifestyle changes. These educational sessions can be held individually or in groups, in the organization of the medical system or of patient organizations, by any health care worker (medical doctor, psychologist, nurse etc.) who is trained in this field. No 2: Patients in need of psychotherapy (e.g., for relaxation, coping strategies etc.) should be offered a psychotherapeutic intervention according to the psychologist’s decision (e.g., CBT, mindfulness) apart from the educational sessions, held by a psychologist. No 3: For those D2TRA patients where according to the psychologist’s judgment a psychotherapy is not sufficient (e.g., depression, fibromyalgia) an examination by a psychiatrist and pharmacotherapy when needed should be offered apart from the psychotherapy and the educational sessions.

According to the physiotherapist’s judgment, every D2TRA patient can be enrolled in one of the following two intervention categories. No 1: Independent regular aerobic and strengthening exercises at home for patients who do not need any supervision or help according to the physiotherapist. A digital or written material for the structuration of these exercises, and the writing of an exercise diary until the next reassessment is suggested in this group of patients. No 2: Regular supervised aerobic and strengthening exercise sessions should be offered to those patients, who need supervision due to compliance problems, disabilities, joint destructions, comorbidities etc.

Additionally to the above mentioned two elements, other NPT options can be offered to D2TRA patients, according to the decision of the rheumatologist/psychologist/physiotherapist/rehabilitation specialist/orthopedist. These NPT options can be additional exercise therapies (e.g., mobilization techniques, special trainings like dance, yoga), physiotherapies (e.g., US, laser, TENS, cryotherapy), balneotherapy, assistive devices, ergotherapy/occupational therapy.

## Discussion

With the new biological and targeted therapies, complete remission has become an attainable goal in rheumatoid arthritis, nevertheless a part of RA patients are still not meeting treatment goals according to treat to target principles, and can be defined as difficult-to-treat RA. The different non-pharmacological therapies (NPT) cover a wide range of interventions, including many exercise modalities, psychological interventions, physio- and balneotherapy, dietary interventions, education etc. NPTs with their complex action can have a synergistic, additive effect with targeted pharmacological therapies, which is highly needed in D2TRA patients, where pharmacological treatment options are often limited. In this review we are summarizing the literature about NPT in RA focusing on patient groups with high probability of D2TRA. A limitation is that the term D2TRA is fairly new, and some studies about NPT were conducted before the widespread use of biological therapies, or patients in many studies were not selected according to the DMARD use, thus trials focusing definitely on D2TRA are yet lacking. This review is also meant to raise attention to this important issue.

We are suggesting that simultaneously with pharmacotherapy, a complex non-pharmacological treatment program should be offered to all D2TRA patients, including regular assessment by a psychologist and a physiotherapist in parallel with the rheumatologist’s investigations, and obligatory and optional NPT elements. Our NPT proposal is focusing on point 9 of the EULAR points to consider in D2TRA (8), which emphasizes the possibility of non-pharmacological interventions to optimize management, and it also has common elements with some other points of the EULAR point to consider: These are the possibility of misdiagnosis or coexistence of mimicking diseases (point 1), importance of shared decision-making (point 4), appropriate education and support (point 10) and the offering of self-management programs, education and psychological interventions (point 10). We are suggesting that following this NPT plan, D2TRA patients can be helped in reaching a better quality of life, better self-management and attainment of treatment goals. Additionally, as treatment options are often limited in D2TRA patients, and non-pharmacological interventions are generally shown to be safe and well tolerated, there is an unmet need for further investigation about different NPT in D2TRA, to clarify their impact on inflammation, cytokine patterns, the neuro-hormono-immunological system, and further clinical studies are needed in the future to optimize the non-pharmacological treatment options in D2TRA, promoting the holistic, personalized treatment approach of this complex disease.

## Author contributions

JM and GN contributed to conception and design of the study. JM, NC-N, GB, TB, and GN did the literature search and wrote the manuscript. All authors contributed to manuscript revision, read, and approved the submitted version.

## Supplementary material

Supplementary Table 1 Summary of literature for exercise therapy in D2TRA.

Supplementary Table 2 Summary of literature for psychological interventions in D2TRA.

Supplementary Table 3 Summary of literature for physiotherapy, balneotherapy and other non-pharmacological interventions in D2TRA.
