# Supplementary material for: Non-pharmacological treatment in difficult-to-treat rheumatoid arthritis
Source: Front Med (Lausanne). 2022 Aug 29;9:991677. doi: 10.3389/fmed.2022.991677 (PMC9465607; doi:10.3389/fmed.2022.991677)
Supplement: Supplementary Table 1 — Summary of literature for exercise therapy in D2TRA. [file Table_1.pdf]

**Reference, type of study**	**Intervention examined**	**Outcome measures examined**	**Patient group examined**	**Results**
Andonian 2021, cross-sectional + intervention study	high intensity interval training (HIIT): alternate bouts of near-maximal intensity aerobic exercise with bouts of lower intensity (supervised treadmill walking exercise sessions three times per week for 10 weeks)	Skeletal muscle gene expression profiling from muscle needle biopsie specimens (m. vastus lateralis), DAS28, pain VAS, physical activity (wearable accelerometer), body composition (abdominal and thigh CT), cardiorespiratory fitness (graded treadmill exercise testing), body composition (BodPod®)	cross-sectional N=47 RA patients, HIIT intervention N=12 RA patients	In the absence of physical activity, RA inflammatory disease activity is associated with transcriptional remodeling of skeletal muscle metabolism. Following exercise training, the greatest improvements in disease activity occur in older, more inflamed, and less fit persons with RA.
Bobos 2019, SLR + meta-analysis	effectiveness of joint protection interventions (JP) in RA and hand osteoarthritis	pain, hand function, and grip strength	3 RCTs for hand OA and in 14 RCTs for patients with RA.	Evidence of very low to low quality indicates that the effects of JP programs compared with usual care/control on pain and hand function are too small to be clinically important at short-, intermediate-, and long-term follow-ups for people with hand arthritis
Brady 2021	investigate associations between PA and sedentary time (ST) during Covid19 pandemic. Different types of physical activity	online questionnaire measuring PA (NIH-AARP Diet and Health Study Questionnaire), ST (International Physical Activity Questionnaire-Short Form), pain (McGill Pain	345 RA patients	Light PA (LPA) was negatively associated with mental fatigue, depressive symptoms, and positively with vitality. Walking was negatively related to physical fatigue and depressive symptoms and positively with vitality. Exercise was negatively associated with physical and general fatigue and depressive symptoms. ST was positively associated with physical fatigue.

		Questionnaire and Visual Analogue Scale), fatigue (Multidimensional Fatigue Inventory), depressive and anxious symptoms (Hospital Anxiety and Depression Scale), and vitality (Subjective Vitality Scale)		
Burghardt 2019, SLR	to determine the specific effects of PA on systemic levels of interleukins and inflammatory markers.	all types of proinflammatory interleukins (IL), CRP, ESR.	RA patients, 15 papers (14 different study populations)	At present, there is no sufficient evidence to conclude that PA has a significant impact on systemic levels of inflammatory markers in RA.
Cheng 2020, Data analysis from National Health Insurance Research Database (2000-2006)	determine whether increased frequency of rehabilitation is protective against stroke or not	diagnosis of stroke. + many comorbidities and medications.	16.224 RA patients, 663 stroke.	Significant positive protective effect against sroke of more frequent rehabilitation sessions could only be demonstrated in RA patients with moderate disease activity.
Cramer 2013, SLR	yoga as an ancillary intervention in rheumatic diseases	For inclusion, RCTs had to assess at least one primary outcome, i.e. pain intensity or function. Secondary outcomes included quality of life, psychological distress and safety of the intervention.	8 RCT (not all for RA)	In two RCTs on FM syndrome, there was very low evidence for effects on pain and low evidence for effects on disability. Based on two RCTs, very low evidence was found for effects on pain in RA. No evidence for effects on pain was found in one RCT on CTS. No RCT explicitly reported safety data.

Cramp 2013, SLR	Non-pharmacological interventions to manage subjective fatigue. (6 types of intervention: Physical activity interventions, psychosocial interventions , herbal medicine , omega-3 fatty acid supplementation, Mediterranean diet , reflexology , the provision of Health Tracker information.)	self-reported fatigue	Total 24 studies until 2012, 2882 RA patients	Physical activity and psychological intervention were statistically significantly more effective than the control at the end of the intervention period. The others not.
Ellegaard 2019, exploratory RCT	whether hand-exercise therapy as an add on to a compensatory intervention program (CIP) will improve the observable performance of activities of daily living (ADL). The hand-exercise program addressed range of motion and muscle strength.	Assessment of Motor and Process Skills (AMPS), self-reported disability (Stanford Health Assessment Questionnaire Disability Index, HAQ-DI), overall disease activity (DAS28), grip strength, and pain. Exploratory outcome was disease activity assessed by US.	55 RA patients	ADL improved in both groups, adding hand exercise showed no benefit for ADL but improved grip strength.
Ercan 2022,	All participants walked on the treadmill for 30 minutes at 60-80% of maximal heart rate.	cytokines, growth factors	40 RA, 40 Control	Baseline levels of inflammatory cytokines, irisin, VEGF and klotho were found to be higher in RA patients compared to the control group. In both groups, there was an increase in serum klotho levels after exercise compared to baseline ($p<0.05$), while a decrease in IL1β, TNF-α levels were observed. While serum VEGF level decreased in RA group, it increased in the control group($p<0.05$). Irisin levels decreased in both groups. IL-6 level did not change in the control group, while it increased in RA

				group. A single exercise session had an acute anti-inflammatory effect in RA patients.
Eversden 2007	hydrotherapy or land exercise, 30min once a week for 6 weeks, joint mobility, muscle strength and functional activities	self-rated global impression of change (7 point scale) immediately after treatment, EuroQol health related quality of life, EuroQol health status valuation, HAQ, 10 metre walk time and pain scores at baseline, after treatment and 3 months later	115 RA patients	SUbjective well-being improved more after hydrotherapy, 10m walking time improved longterm after both exercise forms, pain and functional indices did not improve.
Feldthusen 2015, RCT	12 week program according to an individualised self-care plan from a physiotherapist (3-6 personal meetings) : aerobic physical activity 5 or 3 times a week + individual exercises if needed + balancing life activities	Fatigue (VAS, BRAF-MDQ), DAS28, 1-min sit-to-stand (STS) test, Leisure Time Physical Activity Index (LTPAI), pain and stress VAS, Hospital Anxiety and Depression Scale (HADS), EQ-VAS, Arthritis Self-Efficacy Scale-Swedish version (ASES)	70 RA patients (40% on disability)	fatigue reduced for 6 months, leg strength improved, physical activity improved, anxiety decreased, self efficacy improved.
Gautam 2020	yoga	panel of inflammatory cytokines (IL-6, IL-17A, TNF-α, and TGF-β), mind-body communicative markers (BDNF, DHEAS, β-endorphin, and sirtuin) and transcript levels of various genes (IL-6, TNF-α, NFKB1, TGF-β,	66 RA	significant reduction in DAS28-ESR ($p < 0.001$) and improvement seen in the physical health, psychological, social relationships domains ($p < 0.001$) of QOL, except environmental ($p > 0.05$). The yoga group showed downregulation of IL-6, TNF-α, and CTLA4 and upregulation of TGF-β.

		and CTLA4). DAS28-ESR. WHOQOL-BREF questionnaire.		
Hupin 2021, substudy from a prospective study	physiotherapist-guided aerobic and muscle-strengthening exercises for 1 year , followed by instruction to continue the unsupervised physical activity program autonomously in the next year	changes in heart rate recovery (HRR) post-maximal exercise electrocardiogram (ECG)	25 RA patients	Autonome nervous system activity in RA assessed by HRR was improved by guided physical activity. Machine learning allowed to identify predictors of the HRR response: blood pressure response to exercise, low BMI, and muscular strength.
Hurkmans 2009, systematic review of RCTs, Cochrane	dynamic exercise programs (aerobic capacity and/or muscle strength training), frequency at least twice weekly for > 20 minutes,<br>> 6 weeks, under supervision, land or water.	functional ability, aerobic capacity, muscle strength, pain, disease activity or radiological damage	8 studies 1985-2003, total 575 RA patients	mostly low or moderate evidence on the positive effect on aerobic capacity, functional ability. Good safety. In the longer term the maintenance may prove difficult.
Hurkmans 2010, randomized follow-up study	12 months internet-based individualized training (IT) vs. a general training (GT) programme.	Sustainment for another 12 months of physical activity. Functional ability and quality of life.	110 RA patients	Sustained moderate intensity physical activity up to 12 months in both groups 26% & 19%, SIgnificantly higher RAQoL score in the IT group compared to baseline, no significant change in HAQ or QoL.
Lau 2019, RCT	neural mobilization exercises (median nerve, musculocutaneous<br>nerve, femoral nerve, saphenous nerve,<br>and the entire nervous system) vs. gentle joint mobilisation	Rheumatoid Arthritis Impact of Disease (RAID) questionnaire (pain, functional disability assessment, fatigue, sleep, physical and<br>emotional well-being, and coping/selfefficacy), ESR.	RA patients on csDMARD therapy (N=21)	Significantly more beneficial effect on pain and self-efficacy.
Liao 2021, meta-analysis	the effect of exercise on muscle mass gain. Many	effect on muscle mass (lean body mass , appendicular	9 RCTs (1994-	Exercise therapy significantly affected lean body mass, irrespective of the exercise type. Good

	different exercise programs, exercise protocols with a frequency of 2–3 sessions weekly over a total of 20–72 sessions.	lean mass ,skeletal muscle mass index and cross-sectional area).	2020), 381 RA patients	safety, no significant difference between withdrawas in exercise/control groups.
Manning 2014, RCT	„EXTRA" program comprising of four (1-hour) group education, self-management, and global upper extremity exercise training sessions supplementing the first 2 weeks of a 12-week individualized, functional home exercise regimen.	Disabilities of the Arm, Shoulder, and Hand questionnaire (DASH), (primary outcome measure), the Grip Ability Test (GAT), handgrip strength, Quality of life (RAQoL questionnaire, the Arthritis Self-Efficacy Scale (pain, function, and symptoms subscales), DAS28, participant-reported pain, fatigue, morning stiffness, assessor-rated disease activity. Assessment at 0, 12 and 36 weeks.	108 RA patients	improved upper extremity disability, function, handgrip strength, and self-efficacy in people with RA, with no adverse effects on disease activity.
McKenna 2021, feasibility pilot study	walking-based intervention consisting of 28 sessions, spread over 8 weeks (2-5 times/week), with 1 per week being supervised by a physiotherapist, or to a control group who received verbal and written advice on the benefits of exercise	physical activity (accelerometer), Pittsburgh Sleep Quality Index (PSQI), sleeping pattern measured by the National Sleep Foundation's (NSF) Sleep Diary, pain VAS, Profile of Mood States questionnaire (POMS), Quick Inventory of Depressive Symptomatology	10+8 RA patients	improvements in sleep duration and sleep quality compared to the control group

		(QIDS-SR16), State Trait Anxiety Inventory (STAI), HAQ, CDAI, EuroQoL, fatigue ((BRAF-NS), Exercise Benefits and Barriers Scale (EBBS).		
Metsios 2008 systematic review	effectiveness of exercise interventions on cardiovascular risk profile in RA		1974 to December 2006	No studies were found investigating exercise interventions in relation to cardiovascular disease in RA.
Metsios 2009, cross-sectional study	level of physical activity (International Physical Activity Questionnaire)	blood pressure, cholesterol, low-density lipoprotein , homeostasis model assessment, type-I plasminogen activator inhibitor antigen , tissue-type plasminogen activator antigen, homocysteine, fibrinogen, apolipoprotein B, von Willebrand Factor	65 RA patients	physically inactive RA patients have significantly worse CVD risk profile
Mudano 2019, systematic litterature review	Tai-Chi	benefits (ACR improvement criteria or pain, disease progression, function, and radiographic progression), and harms (adverse events and withdrawals)	seven trials with 345 RA patients	Uncertain benefits
Niedermann 2011, RCT	joint protection: compared individual conventional JP education (C-JP) with PRISM-based JP education (PRISM-JP)- an interactive hands-on-tool. four JP education sessions	JP behaviour, Arthritis Self-efficacy, JP self-efficacy, Hospital Anxiety and Depression Scale, grip strength, hand pain (VAS),	53 RA patients	Better JP behaviour, Arthritis Self-efficacy, JP self-efficacy , less hand pain.

	over 3 weeks, with assessments at baseline, 3 months, 12 months.	DAS28, quality of life (EUROHIS-QUOL 8)		
Romanowski 2020, small, randomized clinical pilot study	Knee joint and patella postisometric relaxation and joint mobilization (10 sessions of 25 min) vs standard exercise.	HAQ, pain VAS, Oxford Knee Score (OKS), Knee Society Score (KSS).	46 RA patients with knee pain	No statistically significant difference in HAQ or knee sores, but significant decrease in pain.
Santos 2019, umbrella review	non-pharmacological non-surgical interventions of any form, duration, frequency and intensity, alone or in combination	pain, functional disability, fatigue, emotional well-being, sleep, coping, physical well-being and global impact of disease	8 Systematic reviews referring 91 RCTs and 9 observational studies. Total 6470 RA patients	Positive effects of multicomponent or single exercise/physical activity interventions, psychosocial interventions and custom orthoses, but only exercise/physical activity interventions appeared to be effective in reducing the global impact of disease and quality of life.
Shadick 2019, prospective cohort study (over 10 years)	The Impact of Exercise, Lifestyle, and Clinical Factors on Perceived Cognitive Function in Patients with Rheumatoid Arthritis.	self-reported memory, concentration, and word-finding difficulties, body mass index (BMI), sleep, depression (Mental Health Index-Depression), DAS28-CRP	1219 RA patients	RA patients were less likely to report word-finding difficulties, poor memory, and concentration as "often" if they were physically active.
Sieczkowska 2021 systematic review and meta-analysis	Home based physical activity vs center-based interventions or no PA.	quality of life, pain, functional capacity, disease activity and inflammation	adults with autoimmune rheumatic diseases (RA, SLE, IIM, SSc, SPA…)	Home-based physical activity improved quality of life and functional capacity, reduced disease activity and pain compared to the non-physical activity control condition. Home-based physical activity interventions were as effective as centre-based interventions for all investigated outcomes.

Silva 2010, SLR Cochrane	To assess the effectiveness and safety of balance training (proprioceptive training) to improve functional capacity.			No studies found investigating the effects of balance training alone or in combination with other therapies in patients with rheumatoid arthritis.
Siqueira 2017, RCT	compare the effectiveness of land-based (LB) and water-based (WB) aerobic exercises, 16 weeks, 3 times per week.	Muscle strength , DAS-28, HAQ, total body densitometry for body composition.	82 RA female	significant improvement in disease activity and functional ability in the WB after 8 and 16 weeks. No significant change in muscle strength or body compocition in the two groups.
Sobue 2021, metaanalysis	Different exercise therapies in RA patients. Analyzed exercise therapies were diverse, differing in target population, intervention method, and duration.	patient-reported outcomes (PROs): HAQ, pain, global disease activity, patient satisfaction, SF-36, EQ-5D, questionnaires (the Michigan Hand Outcome Questionnaire (MHQ) and the Disability of the Arm, Shoulder, and Hand (DASH) Questionnaire).	six RCTs on systemic exercise therapy and three RCTs on upper extremity exercise therapy	Exercise therapy improves patient subjective assessment of pain, physical function, and quality of life (HAQ, pain, SF36). For upper extremity exercise therapy, significant improvements in PROs (Disabilities of the Arm, Shoulder, and Hand Questionnaire, Michigan Hand Outcome Questionnaire) were observed.
Srikesavan 2020, mixed-method, proof-of-concept study	Online hand exercise program for 12 weeks mobility and strength exercises. six exercise training and review sessions, two sessions independently at home	hand pain, hand function, and grip strength, perceived recovery	11 RA	Improvements in grip strength and hand function, no increase in pain. Positive feedback from patients.
Sul 2020, prospective, interventional controlled trial.	12 weeks of upper- and lower-limb strengthening exercise , once-weekly training sessions of 60 min over 12 weeks	hand grip strength , isometric quadriceps contraction, cross-sectional area of the rectus femoris (CSA-RF, UH) , 30 s sit-to-stand test,	35 RA patients	After the 12-week intervention period, the lower-limb strength and the CSA-RF were significantly increased in the exercise group. The activity level did not change significantly in either group. The exercise group exhibited significant improvements in the SF-36 mental health domain scores.

		6MWT, Borg scale score after the 6MWT, quality of life (SF-36 Korean version)		
Summers 2019, cross-sectional comparison	Level of physical activity (PA) and sedentary behaviour (SB) between female RA patients with low disease activity vs those with active arthritis and non-RA controls.	PA was assessed using the ActiGraph accelerometer to determine step count and time spent in moderate-to-vigorous physical activity (MVPA), light activity and sedentary time	40+32 RA patients	RA patients who had long-term disease suppression were more physically active with less SB compared to RA patients with active disease. They had similar light PA and SB to controls although lower MVPA - moderate-to-vigorous physical activity.
Wen 2021, meta-analysis of RCTs	resistence exercises, duration: 3 weeks to 6 months, qiadriceps/leg/grip/arm…	DAS-28, ESR, tome of 50ft walking, VAS, HAQ.	17 RCTs, RA patients 512 treated 498 control.	Resistance exercise showed reducing DAS-28 score, ESR score, and the time of 50 ft. walking. No sign difference in VAS and HAQ.
Williams 2018, Cochrane review	hand exercise versus any non-exercise therapy, most studies involved home programs	benefits and harms of hand exercise: function, strength, pain,	seven studies involving 841 RA people	minimal improvement of hand function, no significant improvement of pain, little or no benefit on mean grip strength or pinch strength. Strategies for adherence possibly beneficial. No adverse effect.
Ye 2020, SLR and meta-analysis	Evaluate the efficacy of yoga for patients with RA	pain, physical function, disease activity, inflammatory cytokines, and grip strength	Ten RCTs including 840 RA patients	Significant effect on physical function, disease activity, grip strength. No effects were found for pain, tender joints, swollen joints count or inflammatory cytokines (i.e., CRP, ESR, IL-6, and TNF-α)
