# Supplementary material for: Non-pharmacological treatment in difficult-to-treat rheumatoid arthritis
Source: Front Med (Lausanne). 2022 Aug 29;9:991677. doi: 10.3389/fmed.2022.991677 (PMC9465607; doi:10.3389/fmed.2022.991677)
Supplement: Supplementary Table 2 — Summary of literature for psychological interventions in D2TRA. [file Table_2.pdf]

Reference, type of study	Intervention examined	Outcome measures examined	Patient group examined	Results
Anvar, 2018 RCT	Six-week arthritis self-management program.	Visual Analogue Scale, Arthritis Self-Efficacy Scale, and self-reported questions regarding mobility	40 RA older women (60-87)	Significant improvements in self efficacy for functional ability and pain management were found in the intervention group compared to those before the intervention and the control group.
Barlow 2000 RCT	six weekly sessions, interactive, multi-component. Topics include: information about arthritis, an overview of self-management principles, exercise, cognitive symptom management (e.g. distraction, visualization and guided imagery), dealing with depression, nutrition, communication with family and health professionals, and contracting. The last of these	arthritis self-efficacy, health behaviours (exercise, cognitive symptom management, diet and relaxation) and health status (pain, fatigue, anxiety,	544 people with arthritis (not only RA), 12-month follow-up	At 4 months follow-up, the ASMP had a significant effect on arthritis self-efficacy, pain subscales, health behaviours (cognitive symptom management, communication with physicians, dietary habit, exercise and relaxation), significantly less depressed and had greater positive mood. In addition, trends towards decreases on fatigue and anxiety. Physical functioning, pain and GP visits remained stable at 4 months. A similar pattern of findings was found at 12 months, furthermore, a significant improvement was found on pain and visits to GPs had decreased.

	involves the setting of realistic goals to be achieved during the forthcoming week.	depression and positive affect)		
Barsky 2010 RTC	RA patients were randomized to cognitive-behavior therapy (CBT), relaxation response training (RR), or arthritis education (AE). All treatment was conducted in groups.	immediately after treatment and 6 and 12 months later: role impairment, and psychological distress, joint examination, erythrocyte sedimentation rate, grip strength, and walking time	168 RA patients	Pain improved significantly at 12 months in the RR and AE groups and showed a nonsignificant positive trend with CBT. Other RA symptoms improved significantly with CBT and AE and showed a nonsignificant trend with RR. There were no significant differences in the outcomes across the 3 treatment groups. When the results for all 3 groups were aggregated, significant benefits were found for pain, other RA symptoms, self-care activities, and social activities.
Davis, 2015	This study compared the impact of cognitive-behavioral therapy for pain (CBT-P), mindful awareness and acceptance treatment (M), and arthritis education (E) on day-to-day pain and stress-related changes in cognitions, symptoms, and affect among adults with rheumatoid arthritis (RA).	At pre- and post-treatment, participants completed 30 consecutive evening diaries assessing that day's pain, fatigue, pain-related	143 RA patients	M yielded greater reductions than did CBT-P and E in daily pain-related catastrophizing, morning disability, and fatigue, and greater reductions in daily stress-related anxious affect. CBT-P yielded less pronounced declines in daily pain-related perceived control than did M and E.

		catastrophizing and perceived control, morning disability, and serene and anxious affects.		
El Miedany, 2012, RCT	targeted patient education, one-to-one cognitive intervention, person-based joint fitness program.	TJC, SJC, We, CRP + patient reported outcome (PROMs) questionnaire : 11 domains	147 RA	significant greater reduction of disease activity parameters, DAS-28 score, as well as improvement of the patients' adherence to anti-rheumatic therapy. At the 18-month follow-up, both the self-management and cognitive behavioral therapy intervention demonstrated improvement for disease activity.
Ferwerda 2018 RTC	Internet-based cognitive behavioral therapy - cost-effectiveness trial	quality-adjusted life years (QALYs) and costs from a societal perspective	RA	The intervention improved the quality of life compared with usual care.

Fogarty 2015 RTC	standardized mindfulness-based stress reduction (MBSR) intervention (8 week program)	SJC, TJC, CRP, pain index, duration of morning stiffness	51 RA	greater reduction in DAS28-CRP scores compared with the control group immediately after the intervention and at both follow-up points , greater improvements in duration of morning stiffness and pain scores, but not significant difference in swollen joint count and CRP.
Hewlett 2011 , RCT	To investigate the effect of group cognitive behavioral therapy (CBT) for fatigue self-management (six (weekly) 2 h sessions, and consolidation session (week 14)), compared with groups receiving fatigue information alone.	Primary outcome: Multi-Dimensional Assessment of Fatigue scale (MAF, 0–50) and a VAS + many secondary outcome measures on pain, fatigue, function and quality of life.	127 RA	At 18 weeks CBT participants reported better scores than control participants for fatigue impact, both in favor of CBT. Secondary outcomes of perceived fatigue severity, coping, disability, depression, helplessness, self-efficacy and sleep were also better in CBT participants.

Hewlett 2019, multicenter RCT	Cognitive Behavioral therapy (CB) approach (RAFT=Reducing Arthritis Fatigue by clinical Teams using CB approaches), comprising seven sessions, codelivered by pairs of trained rheumatology occupational therapists/nurses. Compared to usual care with Arthritis Research UK fatigue booklet.	Primary 26-week outcome fatigue impact (Bristol RA Fatigue Effect Numerical Rating Scale, BRAF-NRS 0-10)	308 RA	Multiple RA fatigue impacts can be improved for 2 years by RAFT program using group therapies with CB approach. The study found that patients' fatigue impact was reduced by both the RAFT program and usual care at 6 months and 2 years, but patients undertaking the RAFT program improved significantly more than those receiving usual care alone. Differences were seen for improvements in fatigue impact, fatigue coping, emotional fatigue and living with fatigue.
Knittle 2015	A 5-week motivation intervention: education session plus a motivational interview from a physical therapist and two self-regulation coaching sessions from a rheumatology nurse.	Level of physical activity, self-efficacy, autonomous motivation, disease activity, functional status, depressive symptoms and fatigue. Post-treatment and 6-months follow-up	78 RA patients	Significant treatment effects were found for leisure-time physical activity, active days/week, self-efficacy and autonomous motivation. No significant effects were found for disease activity, functional status, depressive symptoms or fatigue.

Lorig 2008 RTC	to test the effectiveness of a mail-delivered, internet-based tailored self-management intervention (SMART) compared to the classic Arthritis Self-Management Program (ASMP) or usual care. SMART interventions were provided in months 0-18 and not reinforced.	disability, pain, depression, role function, global severity, doctor visits, and self-efficacy. Results were assessed at 1, 2, and 3 years.	1090 RA patients (SMART vs usual care) or 394 RA patient (SMART vs ASMP)	SMART, was similarly effective to the classic ASMP, with slightly better results in the first year and a slightly more rapid attenuation over the next 2 years. At 1 year, the intervention group significantly improved in 4 of 6 health status measures and self-efficacy. No significant differences in health behaviors or health care utilization were found. Results suggest that both programs are effective, and that the addition of a mail-delivered program could improve accessibility to arthritis self-management treatment.
Lumley 2014 RCT	Two psychological interventions for rheumatoid arthritis (RA) are cognitive behavioral coping skills training (CST) and written emotional disclosure (WED). Randomisation: a 2 × 2 factorial design to one of two writing conditions (WED vs. control writing) followed by one of two training conditions (CST vs. arthritis education control training).	Patient-reported pain and functioning, blinded evaluations of disease activity and walking speed, CRP. assessed at baseline and 1-, 4-, and 12-month follow-ups.	264 RA patients	The combination of WED and CST does not improve outcomes, no interactions between writing and training; however, both interventions had main effects on outcomes, with small effect sizes. Compared to control training, CST decreased pain and psychological symptoms through 12 months. The effects of WED were mixed: compared with control writing, WED reduced disease activity and physical disability at 1 month only, but WED had more pain than control writing on one of two measures at 4 and 12 months. CST improves health status in RA and is recommended for patients, whereas WED has limited benefits and needs strengthening or better targeting to appropriate patients<br><br>.

Sharpe 2012 RTC	Despite evidence that cognitive-behavioral therapy (CBT) is effective for rheumatoid arthritis (RA), little is known about which components of therapy are most efficacious. The present study compared the efficacy of CBT with cognitive therapy (CT) and behavioral therapy (BT) for patients with RA. Randomisation in one of three active treatments (CBT, CT or BT) or a wait-list control (WLC).	disease activity, joint function, disability and psychological functioning	104 RA	The results showed that participants who received cognitive components had greater improvements in tender joint counts and CRP at post-treatment. Those receiving either BT or CT alone improved more on anxiety than CBT or WLC. At 6 months, the three active treatment groups could only be distinguished on tender joints, which favored CT and CBT
Shigaki 2013 RTC	To test an intervention for improving self-management in rheumatoid arthritis (RA) using an online, cognitive– behavioral, self-management group program (RAHelp), with weekly telephone support. A 10-week program with weekly educational modules for improving self-efficacy in self-management of RA, plus tools for group interaction.	Arthritis Impact Measurement Scales 2, Arthritis Self-Efficacy Scale (ASES), Depression Scale, Quality of Life Scale (QLS), Rapid Assessment of Disease Activity in Rheumatology, Loneliness Scale.	106 RA	Group differences with large and moderate effect sizes (ES) were found immediately postintervention for self-efficacy and quality of life, respectively. At 9 months postintervention, differences in self-efficacy and quality of life remained robust.

Terpstra, 2021, Systematic literature review	Internet-based, therapist-guided cognitive-behavioral therapy (iCBT)		8 trials, 1707 patients	Significant medium to large between-group effects were found for psychological outcomes (depression, anxiety, catastrophizing, self-efficacy) and impact on daily life outcomes (impact on daily life, quality of life), whilst results for physical outcomes (pain intensity, fatigue) were mixed.
Zangi 2012 RCT	10-session mindfulness-based group intervention (Vitality Training Programme - VTP) including a booster session after 6 months-was compared with a control group that received routine care plus a CD for voluntary use with mindfulness-based home exercises.	psychological distress measured by the General Health Questionnaire-20, Self-efficacy (pain and symptoms) and emotion-focused coping (emotional processing and expression) ,pain, fatigue, patient global disease activity, self-care ability and well-being.	68 RA	Significant treatment effects in favor of the VTP group were found post-treatment and maintained at 12 months in psychological distress, self-efficacy, pain and symptoms, emotional processing, fatigue, self-care ability and overall well-being. No significant group differences were found in emotional expression, pain or disease activity.

Zautra 2008 RCT	Participants were clustered into groups of 6-10 participants and randomly assigned to 1 of 3 treatments: cognitive behavioral therapy for pain (P); mindfulness meditation and emotion regulation therapy (M); or education-only group (E), which served as an attention placebo control.	daily diaries and laboratory assessment of pain and mitogen-stimulated levels of interleukin-6 (IL-6)	RA 144 adults	Participants receiving P showed the greatest Pre to Post improvement in self-reported pain control and reductions in the IL-6; both P and M groups showed more improvement in coping efficacy than did the E group. The relative value of the treatments varied as a function of depression history. RA patients with recurrent depression benefited most from M across several measures, including negative and positive affect and physicians' ratings of joint tenderness, indicating that the emotion regulation aspects of that treatment were most beneficial to those with chronic depressive features.
Zhou 2020, SLR and metaanalysis	Mindfulness interventions for RA		6 RCT, 336 patienst	mindfulness interventions can significantly improve pain intensity, depression, and symptoms (DAS28) in patients with rheumatoid arthritis compared with conventional therapy
