# Supplementary material for: Non-pharmacological treatment in difficult-to-treat rheumatoid arthritis
Source: Front Med (Lausanne). 2022 Aug 29;9:991677. doi: 10.3389/fmed.2022.991677 (PMC9465607; doi:10.3389/fmed.2022.991677)
Supplement: Supplementary Table 3 — Summary of literature for physiotherapy, balneotherapy and other non-pharmacological interventions in D2TRA. [file Table_3.pdf]

Reference, type of study	Intervention examined	Outcome measures examined	Patient group examined	Results
	**Physiotherapy**			
Attia 2016	laser acupuncture (904 nm, 100 mW power output, 1 minute irradiation time, beam area of 1 cm2 , total energy per point 6 J, energy density 6 J/ cm2 , irradiance 0.1 W/cm2 , frequency 10000 Hz, dutycycle 100%) 3 days/week for 4 weeks.	antioxidant markers: plasma superoxide dismutase [SOD], glutathione peroxidase [GPx], glutathione reductase [GR], and catalase activities, blood glutathione [GSH] concentration, Serum nitrite and nitrate concentrations, plasma ATP concentration, serum RF, plasma IL-6, CRP, ESR, DAS28.	30 RA, 20 healthy	alleviation of oxidative stress and inflammation (decrease in IL-6, CRP, ESR 1 or 2 hours post treatment), improvment of antioxidant and energy metabolic status, suppression of disease activity in RA (DAS28)
Brosseau 2010, Cochrane review	Transelectrical nerve stimulation (TENS) for RA of the hand. Conventional TENS (C-TENS), acupuncture-like TENS (AL-TENS).	The primary outcomes of interest were relief of grip pain and resting pain intensity, relief of joint tenderness, number of tender joints and patient assessment of disease. The secondary objective was to determine the most effective mode of TENS application in pain control.	Three RCTs, involving 78 patients	Conflicting results: 15 minutes of AL-TENS a week, for 3 weeks – decrease in rest pain but not in grip pain, improvement in muscle power. C-TENS resulted in no clinical benefit on pain intensity compared with placebo. However C-TENS resulted in a clinical benefit on patient assessment of change in disease over AL-TENS.

Casimiro 2002, Cochrane review	Underwater continuous ultrasound of the hand.	pain score, grip strength, circumference of proximal interphalangeal (PIP) joints, articular index, and range of motion, level of activity, duration of morning stiffness, number of swollen joint, number of painful joints, degree of wrist dorsal flexion.	Two studies (n of 80)	1. study: No significant difference was found between groups with regard to pain score, grip strength, circumference of proximal interphalangeal (PIP) joints, articular index, range of motion, or level of activity.<br>2. study: increased grip strength, borderline significant effect on the duration of morning stiffness, number of swollen joint, number of painful joints, and on the degree of wrist dorsal flexion.
Drewes 2020, pilot study	short-term transcutaneous non-invasive vagus nerve stimulation (n-VNS) applied to the cervical vagus nerve (self-stimulation on both sides three times per day for 4 days	DAS28-CRP, resting CVT (cardiac vagal tone), cytokines: Interferon-γ (IFN-γ), IL-10, IL12p70, IL-13, IL-1β, IL-2, IL-4, IL-6, IL-8, and TNF-α.	RA high disease activity (n = 16), low disease activity (n = 20)	Well tolerated.<br>High diseases activity: reductions in DAS28-CRP , CRP , and interferon-γ.<br>Low disease activity: no effect on DAS28-CRP, decrease in cardiac vagal tone and a reduction in interleukin-10.
Gizinska 2015	2-week rehabilitation program in two groups: whole body cryostimulation (-10, -60, -110 C) versus traditional rehabilitation (magneto-, electro-, ultrasound and laser therapy)	pain, fatigue, RIM, Health Assessment Questionnaire-Disability Index (HAQ-DI), 50m walking test, DAS28, IL-6, TNFalfa.	25+19 RA patients	Pain, Fatigue, DAS28, RIM, 50m WT, HAQ-DI, IL-6, TNFalfa decreased in both groups. ESR was only reduced in the WBC group, and HAQ-DI showed more reduction in WBC group.
Gok Metin 2013	aromatherapy massage for knees or reflexology for feet vs control, 6 weeks	DAS28, pain VAS, Fatigue Severity Scale	51 RA patients	Pain and fatigue decreased both after aromatherapy and reflexology, eralier in the reflexology group.
Gustavo 2019, secondary analysis of data from a	investigate the dose-response relationship between Neuromuscular electrical stimulation (NMES) and muscle function, and to	quadriceps cross-sectional area and muscle quality were assessed using computed tomography, and strength was measured with	RA, Twenty-four people (48 legs)	Improvement of quadriceps cross-sectional area, muscle quality and strength. Significant associations between NMES training intensity and muscle quality and strength. The minimum NMES

randomized study	establish the minimal NMES training intensity for promoting improvements. 36 NMES treatment sessions for the quadriceps muscles over 16 weeks	an isokinetic dynamometer. NMES training intensity was calculated as a percentage by dividing NMES-elicited quadriceps muscle torque by the maximum voluntary isometric contraction.		training intensity for significant gains in muscle function was approximately 15%.
Hirvonen 2017, RCT	effect of cold treatments on the antioxidative capacity, 3 groups: 1 whole body cryotherapy at −110 °C, (2) whole body cryotherapy at −60 °C, or (3) local cryotherapy. 3 times daily for 7 days.	total peroxyl radical trapping antioxidant capacity of plasma (TRAP) which reflects global combined antioxidant capacity of all individual antioxidants in plasma..	60 RA patients with active disease.	Whole-body cryotherapy at −110 °C induced a short-term increase in TRAP during the frst treatment session, the efect was short and the cold treatments did not cause a signifcant oxidative stress or adaptation during 1 week. No change in the clinical activity or inflammatory variables but significant decrease in pain especially in patients treated with cryotherapy at −110 °C.
Jastrzabek 2013	local cryotherapies: nitrogen vapour at -160 C (group I) or cold airflow at -30 C (group II)	Levels of tumour necrosis factor a (TNF-a), interleukin 6 (IL-6), disease activity score (DAS28), and functional variables	40 RA patients	In both groups: decrease of TNFalfa level, improvement in DAS28, pain, duration of morning stiffness, fatigue, HAQ, active range of knee extension, time, and the number of steps in the 50-m walk test.
Király 2017, RCT	underwater con-tinuous ultrasound therapy to both wrists and hands for 7 min per session with an intensity of 0.7 W/cm2 for 10 sessions.	ESR, CRP, DAS28, pain on a visual analog scale, hand function (fist making, wrist extension and flexion, hand grip strength) and quality of life (Health Assessment Questionnaire [HAQ])	RA patients, 25 treated + 23 control	decrease in CRP, pain, improved left wrist extension, + non-significant decrease in DAS28.

Meireles 2010, RCT	low-level laser in contact with the skin on wrist, MCP and PIP joints, twice a week for a period of 2 months (16 sessions). Aluminum gallium arsenide laser (a wavelength of 785 nm, dose of 3 J/cm2 and mean power of 70 mW) vs. placebo, double blinded.	pain VAS, HAQ, Disabilities of the Arm, Shoulder and Hand (DASH), inflammation - Likert scale, RIM, grip strength, pinch strength, Dexterity (O'Connor test), range of motion (manual goniometer), joint perimetry, patient satisfaction	82 RA patients	No significant effect compared to placebo.
Pelland 2002 Cochrane	electrical stimulation (ES) for improving muscle strength and function in RA, study on hand function in general and on the performance of the first dorsal interosseous muscle in particular	pinch strength and muscle endurance	only one RCT, including 15 RA patients	no clear evidence for the inclusion of ES in the management of RA. Moderate quality evidence on improvement of hand muscle strength and fatigue resistance. Side effects of the ES application were not reported.
Peres 2017, systematic review	combined use of both physical activity and cryotherapy in RA patients	at least present one measure of the aerobic capacity, disease activity or pain relief.	4 studies	Physical activities combined with cryotherapy studies showed an improvement in the disease activity and pain relief, no details of the physical activity or aerob capacity.
Piva 2019, randomized pilot study	Neuromuscular Electrical Stimulation (NMES) versus high-intensity volitional resistance training. 36 sessions over 16 weeks.	muscle structure and function (quadriceps muscles area, density, and strength), physical function (performance-based and patient-reported), feasibility (pain, disease activity, attrition, and adherence), and myocyte characteristics	31+38 RA patients	Both groups experienced significant improvements in muscle structure and function. Both were well tolerated.

		(area, proportion of type I or II muscle fibers, and intramyocellular lipid content)		
	**Balneotherapy**			
Codish 2005, DB-RCT (Sukenik az utolsó szerző)	efficacy of home treatment with mud compresses for the hands. five times a week during a 3-week period. true mud com-presses vs attenuated mud compresses.	number of tender and swollen joints, physician global assessment of disease activity, patient global assessment of the severity of joint pain.	45 RA patients	Pain severity improved in both groups. The number of swollen and tender joints and the scores of physician global assessment was improved only in the true mud compresses group.
Franke 2000, RCT	natural Radon + CO_2 bath versus arteficial CO_2 bath, series of 15 baths in each group.	Pain, functional restrictions (Keitel functional test), Arthritis Impact Measurement Scales (AIMS)	60 RA patients	Good short-term effects in both groups, which were lasting for 6 months only in the radon group.
Franke Annegret 2013	radon spa	pain (primary), quality of life, functional capacity, and medication	681 total, 98 RA patients	superiority of radon therapy was found regarding pain relief and analgesic drug consumption
Santos 2016 RCT	spa treatment for 21 days (sulphur bath, underwater exercise, underwater jets)	HAQ-DI, VAS pain, fatigue and quality of life, physician VAS Global Health Assessment, DAS28	44 RA patients	HAQ-DI at the end of treatment (21 days) and at the 3 month follow-up improved. VAS pain intensity, quality of life and global health assessment improved after 3 months, but not at 21 days.
Sukenik 1990, SB-RCT	Group I was treated with daily mud packs (heated, derived from Daed sea, on four extremities, neck, and back), group II with daily hot sulphur baths, group III with a combination of mud packs and	RIM, 15m WT, grip strength, PIP circumferences, activities of daily living on a scale of 1-6, patient assessment of disease	40 RA patients	Significant improvement for a period of up to three months was observed in the three treatment groups in most of the clinical indices. No laboratory improvement. Except for three mild cases of thermal reaction there were no side effects.

	hot sulphur baths, and group IV served as a control group.	severity on a scale of 0-10, the Ritchie index, ESR, RF, Serum amyloid A.		
Sukenik 1992, DB-RCT	Mud pack treatments once a day (heated, derived from Daed sea, on four extremities, neck, and back). Group 1 was treated with the true mud packs and Group 2 with washed out and less concentrated mud packs. Two weeks.	RIM, hand-grip strength, activities of daily living, patient's own assessment of disease activity, number of active joints, Ritchie index.	28 RA patients	significant improvement in Group 1 in most of the clinical indices, lasting between 1 to 3 months.
Verhagen 2017, Cochrane review	Balneotherapy in RA	pain, improvement, disability,<br>tender joints, swollen joints and adverse events	nine studies involving 579 RA patients, until 2014.	Insufficient evidence, mostly no significant effect, some data showig little effect on pain or number of tender joints.
	**Acupuncture**			
Casimiro 2005, Cochrane review	Acupuncture, electroacupuncture **(**a small electrical impulse is added to the needles)	objective and subjective measures of disease activity	2 studies, 84 patients	No effect of acupuncture (1999)<br>With electroacupuncture (1974), a significant decrease in knee pain was reported in the experimental group, 24 hours post treatment, when compared to the placebo group
Lee 2008, SLR	Acupuncture in RA<br>(needle acupuncture with or without electrical stimulation or moxibustion, laser at precise locations for the purpose of therapy or auricular acupuncture)	pain, ACR20, HAQ, DAS	8 RCT	Four RCTs compared the effects of manual or electro-acupuncture with penetrating or non-penetrating sham acupuncture and failed to show specific effects of acupuncture on pain or other. One RCT compared manual acupuncture with indomethacin and suggested favourable effects of acupuncture. Three RCTs tested acupuncture combined with moxibustion, vs conventional drugs and failed to show benefit.

Sato 2009	acupuncture of the knee joint without electric stimulation	FDG-PET, ESR, CRP, pain, knee joint range of motion (ROM), face scale for patient mood, modified HAQ	6 RA patients	VAS, ROM, face scale and MHAQ improved, but no significant change was detected in ESR, CRP, inflammation on FDG PET.
Seca 2018, DB-RCT	Acupuncture of the hand. Treatment of real acupuncture points vs. treatment of sham acupuncture points vs. no treatment. Assessments took place before and 5 min after AC with follow-ups over 4 weeks.	pain (VAS), Pain pressure threshold in the hand, arm strength (AS) and hand grip strength (HGS), HAQ, SF-36, DAS28, ESR, CRP.	105 RA	In real acupuncture group improvement in self-reported pain, pressure algometry, hand grip strength and arm strength, number of tender and swollen joints, HAQ and QoL. In sham treatment only pain improvement. Worsening in no treatment group.
Tam 2007, pilot study	electroacupuncture (EA), traditional Chinese acupuncture (TCA) and sham acupuncture (Sham) in patients with RA. All patients received 20 sessions over a period of 10 weeks.	pain, ACR core disease measures, DAS 28 score and the number of patients who achieved ACR 20 at week 10	36 RA patients	pain score remained unchanged in all 3 groups. The number of tender joints was significantly reduced for the EA and TCA groups. Physician's global score was reduced for the EA group and patient's global score was reduced for the TCA group. All the outcomes except patient's global score remained unchanged in the Sham group.
	**Others**			
Akbar 2017, review	Omega 3 fatty acids in rheumatic diseases	heterogene	20 clinical trials in RA	16 trials exhibited improvements with fish oil supplements in different clinical outcomes
Cameron 2011, Cochrane Review	Herbal therapy for RA	heterogene	22 studies	Evidence from seven studies indicate potential benefits of gamma linolenic acid (GLA) from evening primrose oil, borage seed oil, or blackcurrent seedoil. Three studies compared Tripterygium wilfordii (thunder god vine) to placebo and one to sulfasalazine and indicated

				improvements in some outcomes, one study showed serious side effects.
Egan 2001 Cochrane	hand splints, special shoes	pain, strength, RIM, QoL, swollen joint count, pain on walking and stair climbing, pain free walking time		Wearing wrist splints during work does not have positive effects, but decreases grip strength.<br>No evidence of positive effect of wearing resting wrist and hand splints, but participants preferred use to non-use, and padded resting splints to unpadded ones.<br>One study of special shoes provided evidence of significant benefits. Extra-depth shoes with semi-rigid insoles provided better pain relief than extra-depth shoes alone. Supporting insoles prevented progression of hallux valgus angle but did not affect pain or function.
Garcia-Morales 2020, RCT	dynamic exercise program (DEP) + mediterran diet (MD). 4 groups: (1) MD + DEP (n = 36), (2) DEP (n = 37), (3) MD (n = 40), and (4) control (n = 31)	Health-related quality of life (SF 36), HAQ at enrollment and after 24 weeks	144 RA patients with low disease activity	QoL improved in MD+DEP and DEP groups, physical component and bodily pain improved also in the MD group.
Guan 2020 SLR + metaanalysis	the effect of Vitamin D supplementation in RA	DAS28 score and components	Six studies (n = 438)	Overall significant improvement in the DAS28, TJC + imprvement of VAS in the European ethnic subgroups.<br>TJC and serum vitamin D were improved in the duration ≤ 12 w subgroups, and the VAS and DAS28 in the duration > 12 w subgroup. With a vitamin D dose ≤50,000 IU, only serum vitamin D and TJC improved, and with a vitamin D dose> 50,000 IU, the VAS and DAS28 improved.
Hagen 2009, Cochrane Review	dietery intervention in RA	heterogene	Fourteen RCTs and one CCT,	Fasting, followed by 13 months on a vegetarian diet, may reduce pain. 12-week Cretan Mediterranean diet may reduce pain. 4-week

			with a total of 837 patients	elemental diet vs an ordinary diet no significant differences. The effects of vegan and elimination diets are uncertain. Higher drop-out rates and weight loss in the groups with dietary manipulation indicate that potential adverse effects should not be ignored.
Hawke 2008, Cochrane	effectiveness of custom foot orthoses (not only RA)		3 study involving 231 RA patients	Effective for rearfoot pain in rheumatoid arthritis, unclear for metatarsophalangeal joint pain.
Macedo 2009, RCT	6 months targeted, comprehensive occupational therapy	Canadian Occupational Performance Measure (COPM), HAQ-DI, DAS-28, EuroQol Index, pain, work satisfaction, and work performance, days missed/month	32 RA patients	improvement in all functional outcomes, pain, DAS-28, QoL, and most work outcomes
Pineda-Juarez 2020, RCT	To determine the combined effect of an mediterran diet (MD) and a dynamic exercise program (DEP) on hand grip strength in women with RA.	hand grip strength, HAQ-DI, DAS28, BMI, body circumference,	106 RA women	DEP-MD, DEP and MD groups. DEP increases hand grip strength and an MD decreases weight and waist circumferences, while the combination of DEP and MD improves disability.
Roelsgaard 2019, Cochrane Review	Different interventions for smoking cessation for reducing disease activity in chronic autoimmune inflammatory joint diseases	smoking cessation (no outcome measure of disease activity)	2 studies, 57 smoker RA patients	Neither of the studies individually provided evidence to show benefit of the interventions tested. Only one study reported on adverse effects which were non-serious.
Somers 2021 pilot study	A 12-session group program (90 minutes per session) : pain coping skills training, lifestyle behavioral weight loss intervention + supervised		50 obese RA patients	reductions in weight and waist circumference, improvements in physical functioning, eating behaviors, pain, and self-efficacy for weight control

	exercise sessions 3 times per week			
Steultjens 2004, Cochrane review	Comprehensive occupational therapy	outcome on functional ability, social participation and/or health related quality of life	9 studies	strong evidence for the efficacy of "instruction on joint protection" , limited evidence for improving functional ability , indicative findings for evidence that "provision of splints" decreases pain.
Tuntland 2009, Cochrane review	Assistive devices in RA	heterogene	29 RA patients	Only one RCT found, which compared the use of an eye drop device to a standard bottle, and rpoved less difficulties when using the device to squeeze out drops and getting the drops in the eyes.
